# Gene flow in a pioneer plant metapopulation (*Myricaria* *germanica*) at the catchment scale in a fragmented alpine river system

**DOI:** 10.1038/s41598-022-12172-x

**Published:** 2022-05-20

**Authors:** Sabine Fink, Andrea Hoppler-Wiedmer, Veronika Zengerer, Gregory Egger, Martin Schletterer, Christoph Scheidegger

**Affiliations:** 1 Swiss Federal Institute for Forest, Snow and Landscape Research, WSL, Zürcherstrasse 111, 8903 Birmensdorf, Switzerland; 2 Institute of Geography and Geoecology (IFGG), Karlsruhe Institute of Technology (KIT), Josefstrassse 1, 76437 Rastatt, Germany; 3 Institute of Hydrobiology and Aquatic Ecosystem Management, University of Natural Resources and Life Sciences (BOKU), Gregor-Mendel-Strasse 33, 1180 Vienna, Austria; 4 Tiroler Wasserkraft AG (TIWAG), Eduard-Wallnöfer-Platz 2, 6020 Innsbruck, Austria

**Keywords:** Ecology, Genetics, Ecology

## Abstract

River alterations for natural hazard mitigation and land reclamation result in habitat decline and fragmentation for riparian plant species. Extreme events such as floods are responsible for additional local species loss or population decline. Tributaries might provide refugia and subsequent source populations for the colonization of downstream sites in connected riverine networks with metapopulations of plant species. In this study, we analyzed the metapopulation structure of the endangered riparian shrub species *Myricaria* *germanica* along the river Isel, Austria, which is part of the Natura 2000 network, and its tributaries. The use of 22 microsatellite markers allowed us to assess the role of tributaries and single populations as well as gene flow up- and downstream. The analysis of 1307 individuals from 45 sites shows the influence of tributaries to the genetic diversity at Isel and no overall isolation by distance pattern. Ongoing bidirectional gene flow is revealed by the detection of first-generation migrants in populations of all tributaries as well as the river Isel, supporting upstream dispersal by wind (seeds) or animals (seeds and pollen). However, some populations display significant population declines and high inbreeding, and recent migration rates are non-significant or low. The genetic pattern at the mouth of river Schwarzach into Isel and shortly thereafter river Kalserbach supports the finding that geographically close populations remain connected and that tributaries can form important refugia for *M.* *germanica* in the dynamic riverine network. Conservation and mitigation measures should therefore focus on providing sufficient habitat along tributaries of various size allowing pioneer plants to cope with extreme events in the main channel, especially as they are expected to be more frequent under changing climate.

## Introduction

Riparian habitats along rivers are of major importance for biodiversity worldwide as they offer high species diversity^1^ and many ecosystem functions^2^. Centuries of river alterations for land reclamation have resulted in habitat reduction and fragmentation especially for sessile riparian plant species^3,4^. Extreme events such as large floods are often responsible for local extinction of plant populations^5^ and are likely to increase under changing climate^6–8^. Tributaries might provide refugia and subsequent source populations for the colonization of downstream sites in connected riverine networks^5,9–11^.

Connectivity between tributaries and downstream rivers is especially important for plant species inhabiting the ever-changing dynamic riverine zone, as local loss or population decline is frequent already at yearly returning floods^12^, despite plants being highly adapted to changing environmental conditions^13^. To counteract genetic diversity loss by reduced local density, functional metapopulation networks connecting populations up- and downstream of rivers are necessary^14^.

Studies on genetic diversity have shown the importance of upstream as well as downstream dispersal for riparian species^15^. Many plant species in habitats close to the waterline display seed morphologies suitable for wind and water dispersal^16^, as well as animal-mediated dispersal mechanisms^17,18^. Despite many means of propagation, riparian metapopulations are generally genetically highly structured and e.g. show isolation by distance patterns^11,19–21^. Water mediated dispersal (hydrochory) might enhance connectivity to distant populations downstream^21,22^, but gene flow along catchments can be highly impacted by barriers such as canyons or reservoirs^23^.

Neutral genetic markers allow to investigate if gene flow and therefore functional connectivity is still present along river networks^24,25^, if there is local population decline^26^, or ongoing migration^27^ despite fragmented habitat. This information is crucial to assess the importance of tributaries and the contribution of single populations to genetic diversity further up- or downstream^28,29^.

In this study, we investigate connectivity along the river Isel and its tributaries by analysing genetic diversity patterns for the characteristic and endangered riparian shrub species, the German tamarisk, *Myricaria* *germanica*^13,30^. This catchment offers a high number of populations of the pioneer plant of the dynamic riverine zon^31^ despite natural canyons, many hydro-morphological river alterations in the past (land use and flood protection) as well as barriers linked to hydropower. The analysis of 45 sites with both small and large (more than 30 individuals) populations within the catchment allows to assess the influence of tributaries on the genetic diversity as well as the connectivity between sites. If gene flow between populations persists, the tributaries and the main reach should reveal similar genotype compositions, and only low population differentiation.

As isolation by distance patterns and high genetic structure in riparian metapopulations indicate short distance wind-mediated upstream and downstream dispersal to have a higher impact than long-distance water-mediated dispersal^24^, the highly variable microsatellite markers are applied to reveal the primary mechanism of dispersal and if unidirectional or bidirectional gene flow along the river network is more frequent. Moreover, we determine the presence of migrants and migration rates, as ongoing gene flow would support intact functional connectivity along Isel and its tributaries.

## Results

**Figure 1:**
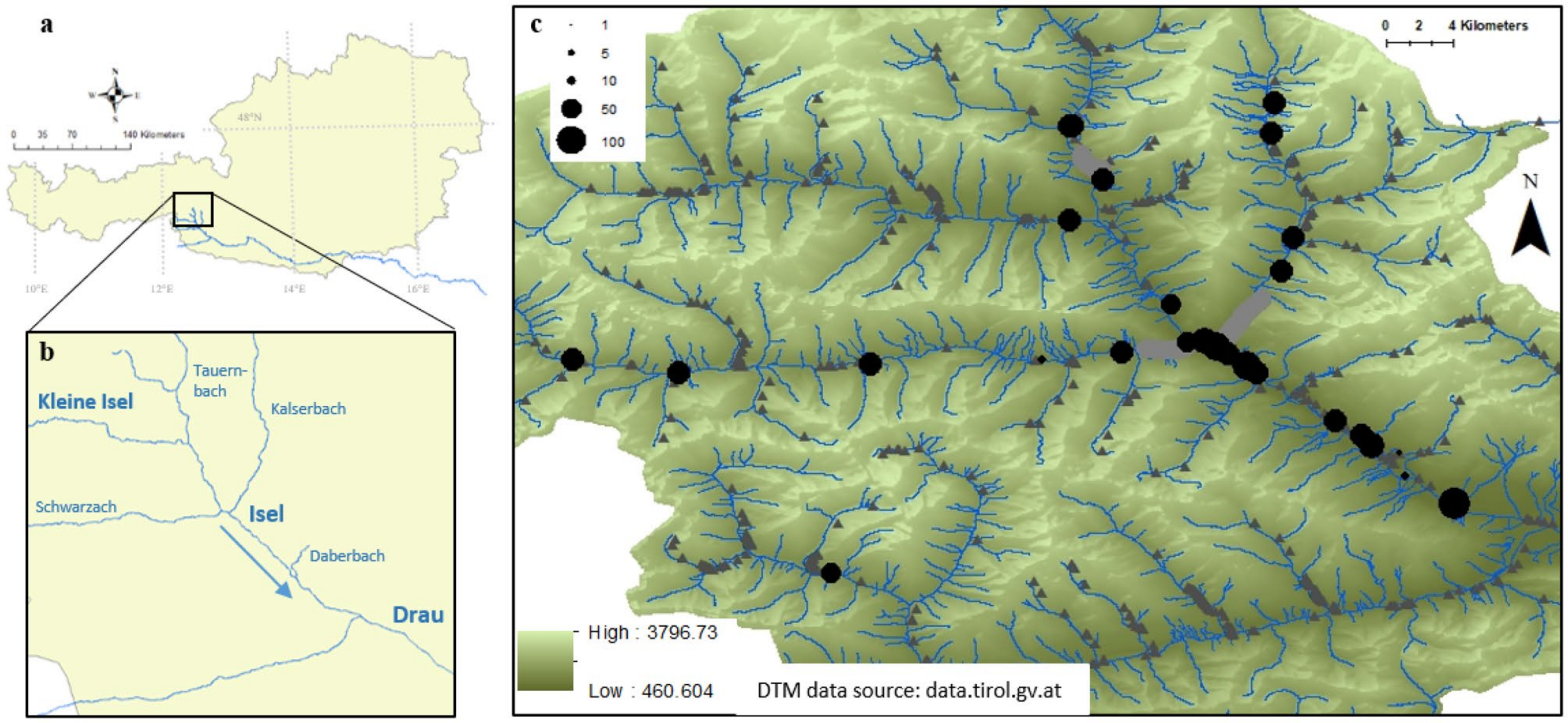
Austria (**a**) with the river Isel and its tributaries in East Tyrol, before they flow into the river Drau (**b**). The sites where *Myricaria germanica* has been sampled along the Tauernbach, Kleine Isel, Schwarzach, Kalserbach, and Daberbach as well as the populations along Isel are shown (**c**): Populations of different size were sampled (size of black dots representative of number of individuals). The river (blue) and riverscape morphology including canyons (light grey river course), dams and embankments (dark grey triangles), and the digital terrain model (DTM from Land Tirol, data.tirol.gv.at) are shown in the background.

A total of 1307 individuals were analyzed from 45 sites (Fig. 1). Sites and populations including age structure were described in detail in the project documentation (see Supplementary Information Table 1 and in^32,33^). Populations showed high numbers of polymorphic loci out of the 22 loci analyzed (Table 1). Private alleles were not found for any population, and genetic diversity analyzed as expected heterozygosity estimates were low for most populations with a maximum of 0.34 for one site at Isel (I-05). Inbreeding coefficients are high for several sites along Isel (I-06: 0.63; I-01: 0.86), Schwarzach (S-08: 0.72; S-04: 0.76, see Table 1). Contrary to that, two sites, one at Isel and one at Schwarzach, have negative F_IS_-values indicating a proportion of outbred individuals (I-04 and S-07 see Table 1).

**Table 1:** Overview of sampling sites in the years 2014 to 2018, and number of samples (n), polymorphic loci, expected heterozygosity (H_E_) per site and inbreeding coefficient of populations (F_IS_).

ID	River	River km**	Year	n	Polymorphic Loci	H_E_	F_IS_
KI-01	Kleine Isel	47,4	2018	37	10	0.297	0.353
KI-02	Kleine Isel	0,4	2018	28	5	0.284	0.286
KI-03	Kleine Isel	38,2	2018	50	8	0.256	0.393
KI-04	Kleine Isel	34,1	2018	14	5	0.258	0.141
KI-05	Kleine Isel	34,1	2018	28	5	0.309	− 0.033
KI-06	Kleine Isel	33,4	2018	20	7	0.202	0.163
KI-07	Kleine Isel	32,0	2018	14	4	0.251	0.216
KI-08	Kleine Isel	30,7	2014	35	10	0.249	0.430
I-01	Isel	24,1–22,5	2014	28	13	0.296	0.864
I-02	Isel	21,6	2018	2	4	0.222	0.200
I-03	Isel	20,2	2018	25	6	0.192	0.497
I-04	Isel	20,1	2018	5	2	0.159	− 0.111
I-05	Isel	19,9–19,6	2014	40	12	0.215	0.313
I-06	Isel	19,8	2018	27	3	0.224	0.625
I-07	Isel	19–18,3	2016	87	15	0.285	0.368
I-08	Isel	17,8–16,1	2016	81	15	0.280	0.326
I-09	Isel	12,1	2016	29	14	0.292	0.412
I-10	Isel	10,8–10,5	2014	31	15	0.335	0.482
I-11	Isel	10,5–9,9	2016	37	14	0.316	0.254
I-12	Isel	7,3	2016	3	10	0.258	0.556
I-13	Isel	5,0–4,6	2016	56	15	0.278	0.300
T-01	Tauernbach	0,0–0,1	2014	31	10	0.253	0.319
T-02	Tauernbach	0,0–0,1	2018	34	11	0.203	0.330
T-03	Tauernbach	5,3 & 5,1	2018	9	11	0.257	0.475
T-04	Tauernbach	2,7–2,5	2014	32	12	0.285	0.350
T-05	Tauernbach	3,0 – 2,6	2018	36	9	0.307	0.336
S-01	Schwarzach	28,6–28,9	2016	31	11	0.288	0.624
S-02	Schwarzach	23,5–23,7	2016	33	11	0.252	0.497
S-03	Schwarzach	14,9–15,0	2014	31	11	0.225	0.448
S-04	Schwarzach	6,7	2016	3	9	0.306	0.765
S-05	Schwarzach	3,3–3,4	2014	31	11	0.288	0.437
S-06	Schwarzach	3,3–3,4	2018	29	9	0.279	0.480
S-07	Schwarzach	n/a	2018	5	2	0.078	− 0.333
S-08	Schwarzach	3,5–3,7	2018	29	5	0.302	0.716
S-09	Schwarzach	0,3	2016	21	11	0.223	0.426
K-01	Kalserbach	16,9–17,0	2016	33	12	0.295	0.471
K-02	Kalserbach	15,8–16,1	2016	31	10	0.258	0.386
K-03	Kalserbach	0,5–0,6	2018	22	0	0.276	0.202
K-04	Kalserbach	7,5 -10,0	2018	42	0	0.332	0.134
K-05	Kalserbach	8,1–8,2	2014	30	13	0.315	0.410
K-06	Kalserbach	6,6–5,9	2014	31	14	0.320	0.444
K-07	Kalserbach	0,2–0,3	2014	29	13	0.320	0.594
K-08	Kalserbach	0,0–0,1	2014	30	12	0.243	0.262
D-01	Daberbach*	0,4	2016	1	4	0.182	0.000
D-02	Daberbach	0,4	2018	24	9	0.281	0.487

**River kilometer according to the Austrian riverine network (Berichtsgewässernetz, https://maps.wisa.bmlrt.gv.at/).

*Small tributary to Isel in the downstram area, analysed as part of the Isel river stretch.

F_ST_-values revealed high values and significant differentiation between most populations (Supplementary Information, Table 2). Of the 44 populations analysed, 12 showed indications for heterozygote excess as analysed under various mutation models and applying different tests in bottleneck (Table 2). Most populations with indication for limited genetic diversity were found at Kleine Isel (4 out of 7 sites), followed by Isel (3 populations), Schwarzach (2 populations), Tauernbach (2 populations) and Kalserbach (1 population, see Table 2).

**Table 2:** Results of test for bottleneck using assumptions of the stepwise mutation model (SMM) and a two-phase model (TPM, combination of 90% of SMM and 10% of IAM) for sites along Isel and its tributaries. p-values of sign test (S-Test), standardized difference test (STD-Test), and one-tailed Wilcoxon sign-rank test (W-Test) as well as the mean expected heterozygosity (mean HE) for der TPM model are given, with significant values (< 0.05) highlighted in bold.

SMM					TPM			
River	Site	S-Test p	STD-Test p	W-Test p	Mean HE	S-Test p	STD-Test p	W-Test p
Kleine Isel	KI-01	**0.01**	**0.00**	1.00	0.24	**0.01**	**0.00**	0.99
Kleine Isel	KI-02	0.48	0.07	0.87	0.23	0.48	0.16	0.84
Kleine Isel	KI-03	0.30	0.14	0.63	0.20	0.30	0.32	0.48
Kleine Isel	KI-04	0.24	0.28	0.72	0.19	0.28	0.37	0.66
Kleine Isel	KI-05	0.08	**0.02**	0.96	0.25	0.34	0.06	0.91
Kleine Isel	KI-06	**0.00**	**0.00**	1.00	0.22	**0.01**	**0.00**	1.00
Kleine Isel	KI-07	0.22	**0.01**	0.95	0.19	0.20	0.04	0.95
Isel	KI-08	0.51	0.48	0.46	0.18	0.52	0.31	0.38
Isel	I-01	0.34	0.41	0.61	0.23	0.58	0.44	0.39
Isel	I-02	na*	na	na	na	na	na	na
Isel	I-03	0.19	0.00	0.96	0.21	0.21	0.00	0.95
Isel	I-04	na	na	na	na	na	na	na
Isel	I-05	0.39	0.20	0.77	0.15	0.41	0.29	0.72
Isel	I-06	0.48	0.32	0.37	0.15	0.44	0.24	0.27
Isel	I-07	**0.03**	**0.00**	0.99	0.22	0.23	**0.04**	0.92
Isel	I-08	0.07	**0.00**	0.98	0.21	0.20	**0.04**	0.96
Isel	I-09	0.30	0.21	0.77	0.23	0.32	0.36	0.64
Isel	I-10	0.50	0.34	0.60	0.28	0.55	0.48	0.47
Isel	I-11	0.19	0.14	0.79	0.25	0.20	0.32	0.64
Isel	I-12	na	na	na	na	na	na	na
Isel	I-13	0.07	**0.00**	1.00	0.21	0.09	**0.03**	0.99
Tauernbach	T-01	0.07	0.19	0.12	0.19	0.06	0.11	0.08
Tauernbach	T-02	0.10	**0.01**	**0.01**	0.21	**0.03**	**0.01**	**0.00**
Tauernbach	T-03	na	na	na	na	na	na	na
Tauernbach	T-04	0.08	**0.02**	**0.01**	0.22	**0.02**	**0.01**	0.00
Tauernbach	T-05	0.48	0.50	0.36	0.25	0.55	0.37	0.34
Schwarzach	S-01	0.18	0.12	0.12	0.23	0.16	0.05	0.09
Schwarzach	S-02	0.53	0.28	0.68	0.19	0.28	0.48	0.58
Schwarzach	S-03	0.25	0.41	0.68	0.16	0.26	0.49	0.55
Schwarzach	S-04	na	na	na	na	na	na	na
Schwarzach	S-05	0.38	0.13	**0.04**	0.23	0.01	**0.07**	**0.00**
Schwarzach	S-06	0.57	0.08	0.79	0.21	0.40	0.18	0.71
Schwarzach	S-07	na	na	na	na	na	na	na
Schwarzach	S-08	0.60	0.16	0.55	0.24	0.59	0.33	0.45
Schwarzach	S-09	**0.01**	**0.00**	0.99	0.15	**0.01**	**0.00**	0.99
Kalserbach	K-01	0.27	0.16	0.19	0.23	0.26	0.08	0.09
Kalserbach	K-02	0.24	0.14	0.12	0.19	0.22	0.09	0.10
Kalserbach	K-03	0.05	0.07	0.87	0.22	0.05	0.12	0.84
Kalserbach	K-04	0.08	0.04	0.96	0.27	0.08	0.15	0.86
Kalserbach	K-05	0.58	0.43	0.39	0.25	0.21	0.39	0.27
Kalserbach	K-06	0.51	0.49	0.50	0.26	0.52	0.30	0.29
Kalserbach	K-07	0.05	0.09	0.86	0.26	0.05	0.24	0.77
Kalserbach	K-08	**0.01**	**0.00**	0.99	0.18	**0.01**	**0.02**	0.97
Daberbach	D-01	na	na	na	na	na	na	na
Daberbach	D-02	0.45	0.42	0.50	0.26	0.42	0.43	0.45

*na for sites with < 10 indviduals.

The results of the AMOVA analysis revealed that the lowest genetic diversity was found between rivers (8.14%, df = 7, Sum of squares = 570.676) and between populations within each river (13.99%, df = 38, Sum of squares = 600.508). Highest variance was within individuals (45.94%, df = 1307, Sum of squares = 1192.5) and within populations (31.93%, df = 1315, Sum of squares = 2748.048).

**Figure 2:**
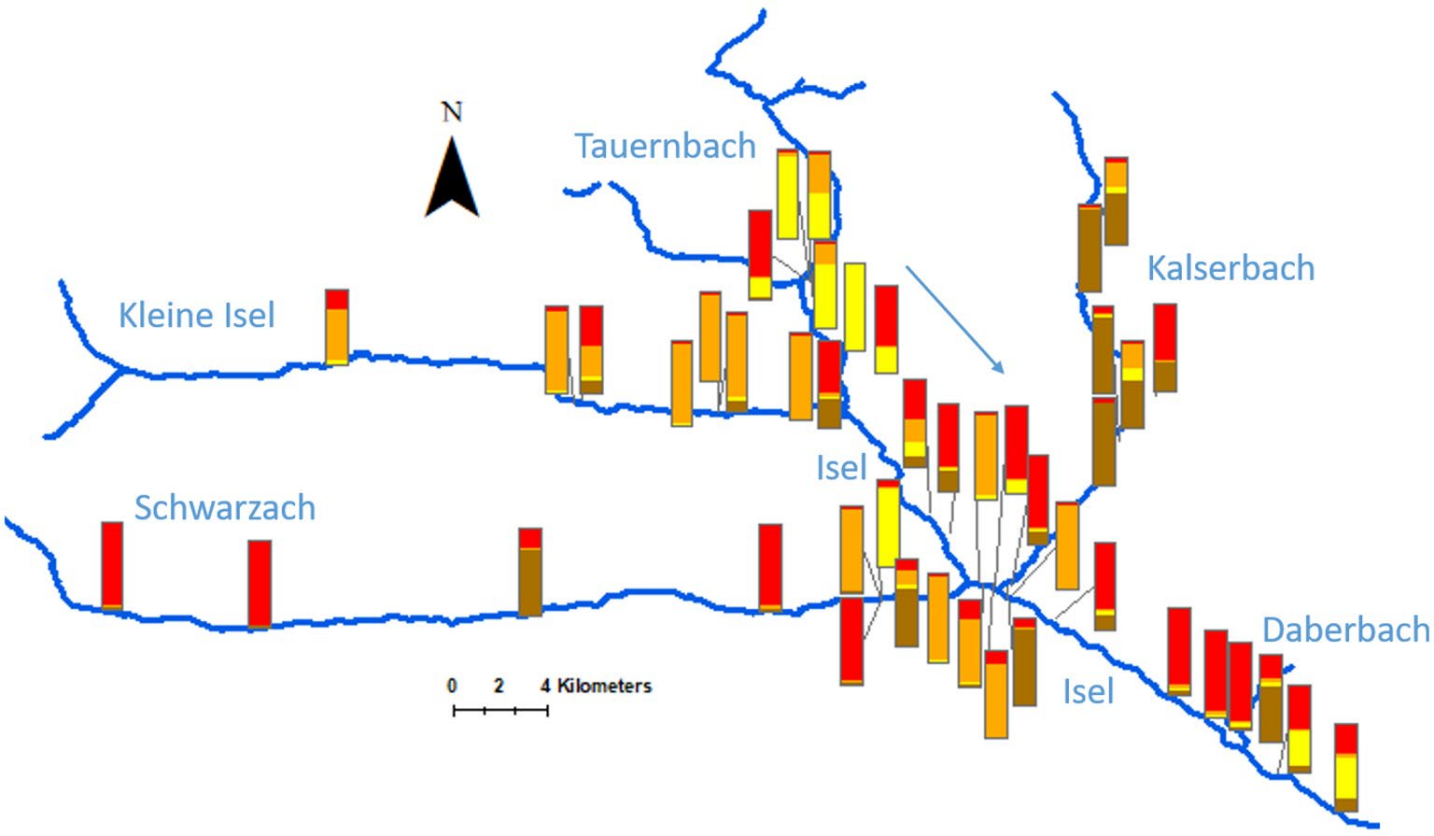
Genetic structure along Isel and its tributaries. For each sampled site, the percentage of genotypes assigned to each of the four groups is shown (displayed in red, orange, yellow and brown, sorted by site see Table 1). The tributaries Tauernbach (north, mainly yellow and red), Kleine Isel (north-west, mainly orange), Schwarzach (west, mainly red) and Kalsersbach (east, mainly brown) all contribute to the high diversity at Isel. The high genetic diversity in the area where Schwarzach and Kalserbach flow into Isel is subsequently lost further downstream, where a majority of genotypes is assigned to one group (red), before the inflow of Daberbach (contribution of yellow group).

The analysis in Structure Harvester revealed that the most likely number of groups of genotypes could be assigned to K = 4 (Supplementary Inforamtion, Fig. 1). The resulting genotype assignment at the population level revealed some gradients, with considerable changes in genotype group assignment of Kleine Isel, Tauernbach and Kalserbach compared to Isel river (Fig. 2, details for values see also Supplementary Information Table 3), but no unique gene pool was found for any river. By comparing genetic differentiation (F_ST_) and distances along rivers, no isolation by distance pattern was detected (correlation between pairwise F_ST_ and geographic distance, R^2^ = 0.0176, Mantel test, *p* = 0.6).

**Table 3:** Detection of first-generation migrants in populations along Isel and its tributaries, and their most probable upstream and downstream source populations (*p* < 0.01). KI = Kleine Isel, I = Isel, T = Tauernbach, S = Schwarzach, K = Kalserbach, D = Dabernbach.

Individuals sampled		Immigrant and sources		
Site	River	Total	Upstream	Downstream
KI-01	Kleine Isel	2		1 × K, 1 × I
KI-02	Kleine Isel			
KI-03	Kleine Isel			
KI-04	Kleine Isel			
KI-05	Kleine Isel			
KI-06	Kleine Isel			
KI-07	Kleine Isel	1	1 × T	
KI-08	Isel	3		3 × I
I-01	Isel	4	4 × T	
I-02	Isel			
I-03	Isel	1	1 × T	
I-04	Isel	1	1 × KI	
I-05	Isel	3	2 × K	1 × I
I-06	Isel			
I-07	Isel	6	4 × K	2 × I
I-08	Isel	6	2 × K, 2 × S, 1 × T	1 × I
I-09	Isel	3	2 × I	1 × I
I-10	Isel	4	1 × I, 1 × K	2 × I
I-11	Isel			
I-12	Isel	1		1 × I
I-13	Isel	4	2 × K	2 × I
T-01	Tauernbach	2		1 × I, 1 × T
T-02	Tauernbach	6		6 × T
T-03	Tauernbach			
T-04	Tauernbach	1		1 × I
T-05	Tauernbach			
S-01	Schwarzach	5		5 × S
S-02	Schwarzach	1		1 × I
S-03	Schwarzach			
S-04	Schwarzach	1		1 × I
S-05	Schwarzach	3		3 × I
S-06	Schwarzach	1		1 × I
S-07	Schwarzach	1	1 × T	
S-08	Schwarzach			
S-09	Schwarzach	1	1 × S	
K-01	Kalserbach	4		4 × K
K-02	Kalserbach	1		1 × S
K-03	Kalserbach			
K-04	Kalserbach			
K-05	Kalserbach	1	1 × K	
K-06	Kalserbach	2	2 × K	
K-07	Kalserbach	2	1 × S	1 × I
K-08	Kalserbach	3	2 × S	1 × K
D-01	Dabernbach			
D-02	Dabernbach	4	2 × T, 1 × I	1 × I

**Figure 3:**
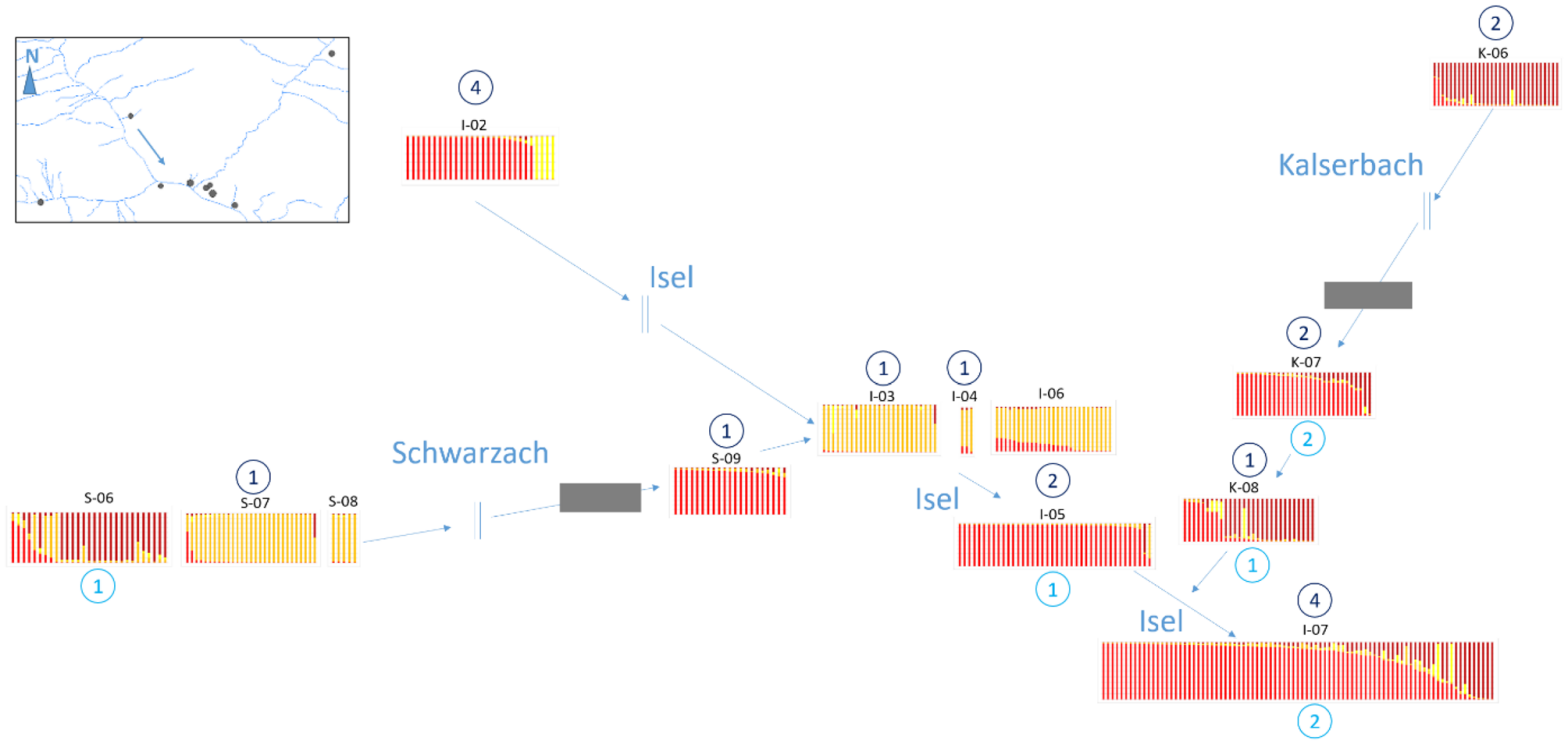
Genetic structure and number of migrants at the junction of Schwarzach (left), Kalserbach (right) and Isel (center) along the rivers (blue lines). The four genotype groups are represented in different colours (red, orange, yellow and brown) and number of immigrants from a source upstream (dark blue) and downstream (light blue) are shown in circles. Although two sites (one at Schwarzach and one at Kalserbach) are separated from the next source population upstream by canyons (grey blocks), they show immigrants from upstream. Geographically close populations next to the inlet of Kalserbach all show migrants from downstream sites.

A total of 80 individuals of the 1307 analyzed were more probable to originate from other populations than they were found in (*p* < 0.01). Of the 45 sites studied, 30 showed individuals which are most likely first-generation migrants from another population (Table 3, *p* < 0.01). Most migrants per site were detected in two population at Isel (I-08 and I-09), and potential sources were traced to upstream tributaries but also to other sites at Isel downstream. Similar to the findings for Isel, sources of migrants were assigned to both up- as well as downstream sites for all tributaries (Table 3 and Fig. 3). We detected mainly of non-significant recent migration rates (Supplementary Information, Table 4). The only significant values > 0.2 were obtained for geographically close populations at Kleine Isel (KI-02, KI-04, KI-05, KI-06, KI-07), as well as the close populations at the junction of Isel and Schwarzach (I-07, I-08, S-09, Supplementary Information, Table 4).

## Discussion

### Genetic diversity of the Isel metapopulation

The German tamarisk displays many populations along the Isel catchment despite changes in river morphology and dynamics by humans (see Fig. 1 as well as in^31^). Our comprehensive study on both large and small populations shows that genetic diversity is especially high at the large populations at the Isel river where also patterns of population demography indicate ongoing rejuvenation.

Contrary to the general situation at the main Isel reach (downstream of Matrei i.O.), many populations of various size within the catchment show high inbreeding. As the German tamarisk is capable of selfing^23^, even large populations might display low genetic diversity due to few founders and genetic drift^34^, similar to other shrub species in dynamic environments^35^. This is apparent in a population at Isel, occurring after the junction with Schwarzach and Kalserbach, which has a long history of German tamarisk presence^31,36^. Despite a large population size, the central position in the riverine network and no barrier limiting dispersal, signs of inbreeding and a bottleneck were detected at this site. This stresses the importance of genetic analyses to assess the comprehensive diversity of a single population^37^, and the full impact of a site to a metapopulation network^5,14^.

Genetic diversity patterns are reflecting habitat fragmentation due to river morphology and connectivity^38–40^, providing valuable insights for conservation measures when linked to demographic and topographic data^41^. In the headwaters (Kleine Isel) and the tributary Tauernbach, detected reduced genetic diversity is reflecting limited connectivity due to topographical characteristics^31,42,43^. Similarly, the signs of bottlenecks at two sites close to the confluence with Isel of Schwarzach (S-09) and of Kalserbach (K-08) are most probably due to the location downstream of canyons, which are responsible for habitat fragmentation and non-functional connection to upstream populations by wind-mediated dispersal (see in^23^). However, the genetic diversity pattern at the catchment revealed that downstream transport via canyons (i.e. by floating seeds or vegetative dispersal by plant ramnets) has been possible through the Kalserbachklamm, the Defregger-Klamm and the Prosseggklamm, similar to findings for other riparian species^44^.

### The role of tributaries on gene flow

Contrary to findings of high differentiation along rivers for other riparian plant species (by diaspore mimics^45^, studies on woody riparian guildes^46^, and studies on floodplain meadow species^47^), the tributaries of Isel do not display unique gene pools or clear isolation by distance patterns, as e.g. found for fish^48^. Still, some tributaries showed higher percentage of certain groups of genotypes (e.g. Tauernbach, see Fig. 2) as well as some gradients along the river (e.g. Schwarzach, west–east gradient following flow direction, see Fig. 2). Similar to findings for *M.* *germanica* populations in other countries^23,49^ as well as other riparian species^20,21^, the studied populations were highly differentiated in pairwise comparisons. This pattern reflects a typical metapopulation for a species with various dispersal mechanisms (see in^24^), which has been found for other riparian plant species in the dynamic riverine zone^50^.

Recent migrants were detected both between large as well as small populations, although contemporary migration rates were small. This indicates that individuals of tributaries might have been sources for long distance dispersal downstream (e.g. by vegetative dispersal) during extreme flood events^24^, despite being isolated under steady-state-conditions (e.g. Daberbach, see Fig. 2). First-generation immigrants (as detected at two sites) can lead to increased genetic diversity in future generations if the habitat is suitable for local species expansions^51^.

Especially interesting for metapopulation studies along rivers are junctions, as they form unique riparian habitats and allow to assess the impact of single inlets on the genetic diversity at the catchment^52^. The genetic structure of populations at junctions can further reveal functional connectivity up- and downstream at a small scale^25^. At the junction of Schwarzach and Kalserbach with Isel, the populations of the tributaries are more related to the populations at Isel downstream than to the other sites at the tributaries upstream, partly also due to the canyons (Fig. 3). With all populations showing at least one migrant in this geographically limited area, the junction is a hotspot of gene flow both up-and downstream, and therefore a focal point in the metapopulation network^53^.

### Directional gene flow and importance of barriers to dispersal

First-generation migrants can reflect vegetative as well as propagule dispersal, as the German tamarisk is capable of both^18^. While vegetative dispersal is unidirectional by water flow, seed dispersal by wind is common for the German tamarisk^54^ and is playing an important role for functional connectivity for many riparian species^15^. The lack of an isolation by distance pattern and the detection of migrants both from upstream and downstream sources suggest bidirectional gene flow mainly by wind dispersal, similar to previous findings^49^. Hydrochory is less likely, given the high number of human-made barriers such as check dams along the Isel catchment, which likely inhibit water-mediated dispersal ^4,23,55^.

Long-distance wind dispersal seems to be rare (but see^15^), but for the German tamarisk it has even been previously detected even between catchments^49^. Additionally, gene flow over long distances is possible also by pollen mediated dispersal^18^, and insect pollinators can cross barriers to both water- and wind-mediated gene flow. The current genetic structure indicates that *M*. *germanica* can partially overcome both natural canyons and man-made check dams along Isel and its tributaries, although limitations in gene flow might only be detectable after a certain time^26^.

Unexpected re-colonization of isolated sites could be linked to human-mediated dispersal by gravel extraction or relocation during or after construction along rivers, as seen at a site within a series of sediment check dams at Ködnitzbach (a tributary to Kalserbach), where a large population was established at the small gravel bar within the highly impacted river stretch K-03, see Fig. 2 and^32^. This is similar to findings of another study after sediment check dam construction^56^. The fate of such populations remains unclear, as subsequent lack of sediment and hydrological dynamics can influence population persistence, e.g. by preventing rejuvenation^57^.

### Management and conservation implications

Due to the lack of a long-term monitoring of the metapopulation at Isel^36^, indirect evidence for ongoing functional connectivity as provided by this study is necessary for management planning for the river stretches within the Natura 2000 area. Although habitat fragmentation is present (e.g. natural canyons, lateral embankments and check dams), the metapopulation network is functionally connected. Still, management strategies are necessary, as *M. germanica* populations are frequently found in dynamic riverine zones, which are subject to major loss during extreme events (such as HQ_30_ or HQ_100_ flood events). As populations with mainly young individuals show less genetic diversity than sites with older plants, the main focus of conservation strategies should be on protecting large populations with diverse age classes. As all tributaries contribute to the gene pool, sheltered and currently more isolated sites (e.g. Daberbach) might provide important refugia and sources for subsequent re-colonization after extreme events as exemplified by^58^.

For management planning, knowledge on species’ dispersal mechanisms is especially important for species inhabiting rivers and riverscapes^59^. Wind-mediated dispersal with a continuous supply of seeds during summer^54^ together with pollen mediated gene flow mainly allow for shorter dispersal distances^60^. Smaller populations or less persistent sites in highly dynamic riverine zones might provide important short-term nodes in the metapopulation network^52^, and currently unsuitable habitat should be restored to provide a better network. The overall habitat loss, e.g. due to hydro-morphological changes like channelization, is the major threat for *M. germanica*, and therefore, re-introduction projects are often initiated in revitalization projects such as river widenings e.g.^61–63^. If human mediated re-introductions of individual plants or seeds are considered, they should only use material originating from the closest populations along the tributary, given the genetic differentiation detected in this study. A future monitoring of sites and population structures as well as changes in genetic diversity would allow to assess the status and development of the German tamarisk along Isel based on the presented study.

### Conclusions

Our study on the genetic diversity of *M*. *germanica* along the Isel and its tributaries provides a first comprehensive overview of the metapopulation, and highlights the importance of tributaries within the catchment for maintaining gene flow. Both large and small populations might play important roles in the metapopulation network, but are equally subject to population declines. Conservation management of the Isel and its tributaries should therefore focus on enabling habitat formation or restoring habitat for the German tamarisk, with a special focus on the migration hotspots at the junctions. This ensures the survival of *M. germanica* under expected more frequent and more intense extreme events such as floods due to changing climate despite fragmented habitat.

## Methods

### Study species

The German tamarisk, *Myricaria germanica* (L.) Desv., is the main indicator for the protected habitat “Alpine rivers and their ligneous vegetation with *Myricaria germanica*”, Natura 2000 code 3230 for a study on the habitat, e.g.^64^. In Tyrol, *M*. *germanica* is protected since 2005 (Tiroler Naturschutzgesetz, Article 23, attachment IV lit.b) and the manipulation of the plant as well as habitat changes resulting in loss of individuals is prohibited (Ordinance of Nature protection, Verordnung der Tiroler Landesregierung 2006, Article 1 and 2). Over the last decade, the species showed a severe decline along European rivers^30,36^. As a typical pioneer species on gravel banks and bars, the German tamarisk is threatened, if sediment and hydrological dynamics are restricted.

The shrubs can reproduce vegetative by re-rooting of branches, but also produce seeds that germinate within 48 h after seed landing^65^. Juvenile plants reach age of first flowering after 1–2 years see also^16,64^, and the sentenced flowers are pollinated by insects^18^. Additionally, the plant is reported to be capable of selfing^23^. As it provides nutrition for insects such as honey bees, recommendations for the cultivation of the shrub along rivers in order to improve the honey yield were promoted in Tyrol in the mid-twentieth century^66^. Seeds of the German tamarisk have a pappus, facilitating both wind- and water-mediated dispersal^43,67^. For wind-mediated dispersal, the majority of seeds were found close to the mother plant, but dispersal kernels show long tails^54^, with rare long-distance dispersal of several kilometers^43^. While water-mediated dispersal is known as long distance dispersal for many riparian plants^68^, dams and canyons are reported to inhibit water-mediated dispersal of *M. germanica*^23^.

### Study site: Isel and tributaries

This study focuses on the river Isel (ca. 50 km) in East Tyrol, Austria, and its tributaries: North to South: Tauernbach (17 km), Schwarzach (43 km) and Kalserbach (17 km) as well as the small stream Daberbach (3 km, Fig. 1). The river Isel is usually referred to as “Kleine Isel” upstream of the junction with Tauernbach and thereafter as “Isel” further downstream. In 2015, the Isel and stretches along the tributaries Kalserbach and Schwarzach (“Osttiroler Gletscherflüsse Isel, Schwarzach und Kalserbach”) were designated as Natura 2000 area (see https://natura2000.eea.europa.eu/Natura2000, site code AT3314000).

In the Isel catchment mean annual temperatures of 0.5–1 °C and precipitation of 1400–1500 mm per year were recorded, with high amounts of snow and ice in Winter^69^. Along the tributaries of the Isel, many constructions to prevent natural hazards such as sediment check dams are established, and there are also some barriers related to hydropower (see Fig. 1). The Isel and its tributaries have been monitored in the past for the presence of *M*. *germanica*^31,36^ and mainly provide habitat for the study species where the shoreline of the river is not stabilized by lateral embankment or where the formation of gravel banks is possible due to wide river section^31^.

The Tauernbach mainly consists of deep canyons upstream and has artificial side banks along the downstream regions, but the stretch below the canyon Prosseggklamm (see Fig. 1) offers habitat to pioneer vegetation including the study species^31^. Similarly, large canyons in the lower course the Kalserbach (Kalserklamm) and the Schwarzach (Defregger Klamm) display German tamarisks upstream and downstream of the canyons (Fig. 1). However, long stretches of these tributaries are also stabilized by lateral embankments, reducing the habitat availability for the German tamarisk. Therefore, the study region covers both sites with large, long-term persistent populations but also more remote sites with few individuals.

### Field work

Field work along Isel and its tributaries was performed from 2014 to 2018, and a total of 45 sites were sampled (Table 1). All individual plants were recorded using GPS (Garmin Oregon 700). To assess the population structure at each site, plants were assigned to four age categories as defined in previous studies^69^. For large populations with over 30 individuals, at least 30 samples per sites were collected, while all individuals were sampled at sites with few shrubs (see Table 1). For each sampled individual, plant leave material was collected, dried with Silica gel (Silica Gel Orange, ROTH, Nr. P077.1) and subsequently stored at − 20 °C. All methods were performed in accordance with relevant guidelines and regulations.

### Genetic analysis

For each sample, 15 mg ± 3 mg leave material was lyophilized (BETA 1-8 L0 plus, Christ, at 40 bar and − 55 °C) prior to total DNA extraction (DNeasy^®^96 Plant Kit, Qiagen, Cat.No. 69181). Following the protocol of^70^ using Multiplex PCR Master Mix», 2x (Qiagen, No. 1066295), 22 microsatellite loci were analysed using PCR. All PCR products were diluted (1:2) with ultrapure water, and 1 µL of the mix was added to 9.5 µL HiDi-LIZ solution (Applied Biosystems, Lot. 1,401,295) and size standard mixture (concentration 15 µl/mL, GeneScanTM-500 LIZ^®^, Applied Biosystems, Lot. 1,401,359) for the analysis on a 3730xl DNA Analyzer (ABI, Applied Biosystems).

### Data analysis

Using the software GeneMapper (Applied Biosystems, V5.0), fragment analysis of the 22 microsatellite loci was performed using scoring bin sets of previous studies for details see^23,49,70^. Fragment length raw data is available from the authors upon request. The resulting multilocus genotype data of each individual was formatted and analyzed using the packages “poppr” and “tidyr” in the program R^71^: polymorphic loci, private alleles, the expected heterozygosity as well as the inbreeding coefficient (F_IS_) were calculated to assess genetic diversity. Additionally, the program Arlequin 3.5^72^ was used to calculate F_ST-_values for population differentiation, and pairwise comparisons to detect significantly differentiated populations.

To identify if single populations underwent a drastic decrease in effective population size, we used the program Bottleneck^73^ for populations with at least 10 individuals. We performed all three tests available in Bottleneck, the sign test, standardized difference test^73^ and the one-tailed Wilcoxon sign-rank test^74^ to evaluate if the populations showed a heterozygosity excess or deficit. Expected heterozygosity was based on simulations for the genetic distribution for each populations under the assumption of two models, as the microsatellite motifs did not allow to make a prior choice on a single model^70^: the stepwise mutation model (SMM) and a two-phase model (TPM) allowing for a combination of 90% of SMM and 10% of infinite allele model.

Molecular variance (AMOVA) within and between populations using Isel and each of its tributaries as a predefined geographic structure (resulting in 6 groups) was performed in Arlequin 3.5^72^. The genetic structure of the study site was assessed in the program structure 2.3.4, testing for K = 1–45 groups^75^, with 10^8^ iterations and a burn-in of 10^4^. The output of this Bayesian approach to identify the number of groups the multilocus genotypes could be assigned to was tested for statistical support in the program Structure Harvester^76^.

Limitations to gene flow in the study sites as seen in an isolation by distance pattern was tested by a Mantel test based on the comparison of genetic differentiation (F_ST_) of populations with more than 10 individuals and geographic distances along rivers Manteltest in GenAIEx 6.503^77^. Distances along rivers between the chosen populations were determined using the package “riverdist” in R^71^. To assess if migration between sites is ongoing, we applied a Bayesian approach^27^ to identify migrants using the software GeneClass^78^. In this program, we estimated the likelihood of first-generation migrants by assessing the likelihood of an individual multilocus genotype to originate from the population it was sampled from compared to the likelihood that it is sampled from another population in the catchment see also in^78^. Probability computations (based on Monte-Carlo simulations) were done using the algorithm of Paetkau^79^ with 100′000 simulated individuals and a 0.01 type I error rate.

To analyze the extent of migration between populations, we used the program BayesAss 3.0.4^80^ implementing Markov chain Monte Carlo techniques to simulate recent migration rates from allele frequencies of multilocus genotypes. Following the manual for BayesAss, we first identified suitable mixing parameters for migrations rates, allele frequencies and inbreeding coefficients to allow for acceptance rates between 20 and 40%, as suggested from empirical analyses^81^. We run simulations with 10^8^ iterations and 10^4^ million burn-in, and diagnosed for convergence of chains using the software Tracer 1.7^82^. Runs were repeated with different random seeds and we then identified a suitable run calculating Bayesian Deviance using the R script as described in^83^. To identify significant migration rates, we checked if the 95% Confidence Intervals (CI) excluded 0^84^.

### Ethics approval

All sampling permits were issued by the Office of the Tyrolean Regional Government (Amt der Tiroler Landesregierung). Sampling in 2014 and 2016 was carried out on behalf of the Office of the Tyrolean Regional Government. Sampling in 2018 was carried out based on the permit (for sampling parts of protected plants) NSCH/N-269/6–2017 from 06.09.2017 (district authority Lienz) and the decision of the State Administrative Court of Tyrol LVwG-2017/41/2267–19 from 23.05.2018.

## Supplementary Information

Supplementary Information 1.

Supplementary Information 2.

Supplementary Information 3.
