# Supplementary material for: Gene flow in a pioneer plant metapopulation (Myricaria germanica) at the catchment scale in a fragmented alpine river system
Source: Sci Rep. 2022 May 20;12:8570. doi: 10.1038/s41598-022-12172-x (PMC9122923; doi:10.1038/s41598-022-12172-x)
Supplement: Supplementary file 1 — Supplementary Information 1. [file 41598_2022_12172_MOESM1_ESM.docx]

**Supplementary Information**

to manuscript

**Gene flow in a pioneer plant metapopulation (*Myricaria* *germanica*) at the catchment scale in a fragmented alpine river system**

Sabine Fink^a,b^, Andrea Hoppler-Wiedmer^b^, Veronika Zengerer^b^, Gregory Egger^c,d^, Martin Schletterer^d,e^ and Christoph Scheidegger^b^

^a^ corresponding author: sabine.fink@wsl.ch, phone: +41 44 7392836, fax: +41 44 7392 215,

^b^Swiss Federal Institute for Forest, Snow and Landscape Research, WSL, Zürcherstrasse 111, 8903 Birmensdorf, Switzerland

^c^ Karlsruhe Institute of Technology (KIT), Institute of Geography and Geoecology (IFGG), Josefstrassse 1, 76437 Rastatt, Germany

^d^ University of Natural Resources and Life Sciences (BOKU), Institute of Hydrobiology and Aquatic Ecosystem Management, Gregor-Mendel-Strasse 33, 1180 Vienna, Austria

^e^Tiroler Wasserkraft AG (TIWAG), Eduard-Wallnöfer-Platz 2, 6020 Innsbruck, Austria

**Supplementary Information, Figure 1:**

The program structure harvester revealed the highest Delta K for K=4 groups for tests between K=2 to K=47 (for simplicity, only up to K=9 is shown in figure).

**Supplementary Information, Table 1:**

Field code of sampling sites (no) used during different field studies and for official descriptions of field sites ^1,2^, and the ID used in this study as well as information on the river name, river km and year of sampling.

** river kilometer according to the Austrian riverine network (Berichtsgewässernetz, https://maps.wisa.bmlrt.gv.at/)

*small tributary to Isel in the downstram area, analysed as part of the Isel river stretch

1 Schletterer, M., Gewolf, S., Egger, G. & Fink, S. Forschungsprojekt Tamariske: Genetische Untersuchung von Populationen an der Isel – Dokumentation der Beprobungen 2018. 32 (Innbruck, 2019).

2 Scheidegger, C. & Wiedmer, A. Genetische Untersuchung zur Deutschen Tamariske in Tirol. *Birmensdorf: Eidg. Forschungsanstalt WSL* (2014).

**Supplementary Information, Table 2: see separate Excel file**

**Supplementary Information, Table 3:**

Genotype assignment at the population level by the program Structure using K= 4 groups along Isel and its tributaries. Percentage of individuals’ genotypes according to the K=4 groups assigned to each of the four groups (red, orange, yellow, purple, colours similar to Figure 2) identified in the program structure and number of individuals analysed (n).

**Supplementary Information, Table 4: see separate Excel file**
